# Prevalence and associated factors of suicidal ideation among college students during the COVID-19 pandemic in China: a 3-wave repeated survey

**DOI:** 10.1186/s12888-022-03968-2

**Published:** 2022-05-15

**Authors:** Shun-wei Liang, Li-li Liu, Xiao-dan Peng, Jian-bin Chen, An-di Huang, Xia-yong Wang, Jing-bo Zhao, Fang Fan, Xian-chen Liu

**Affiliations:** 1 Department of Psychology, School of Public Health, Southern Medical University, Guangzhou, China; 2 Mental Health Education and Counseling Center, Guangzhou Academy of Fine Arts, Guangzhou, China; 3 Mental Health Education and Counseling Center, School of Public Health, Southern Medical University, Guangzhou, China; 4 School of Psychology, Center for Studies of Psychological Application, Ministry of Education Key Laboratory of Brain, Cognition and Education Sciences, and Guangdong Key Laboratory of Mental Health and Cognitive Science, South China Normal University, Guangdong, China; 5 Center for Public Health Initiatives, University of Pennsylvania, Philadelphia, PA USA

**Keywords:** Suicidal ideation, Risk factors, Prevalence, COVID-19 pandemic, College students

## Abstract

**Objective:**

The coronavirus disease 2019 (COVID-19) pandemic, a major public health crisis, harms individuals’ mental health. This 3-wave repeated survey aimed to examine the prevalence and correlates of suicidal ideation at different stages of the COVID-19 pandemic in a large sample of college students in China.

**Methods:**

Using a repeated cross-sectional survey design, we conducted 3 online surveys of college students during the COVID-19 pandemic at 22 universities in Guandong, China. The 3 surveys were conducted during the outbreak period (T1: 3 February to 10 February 2020, *N* = 164,101), remission period (T2: 24 March to 3 April 2020, *N* = 148,384), and normalized prevention and control period (T3: 1 June to 15 June 2020, *N* = 159,187). Suicidal ideation was measured by the ninth item of the Patient Health Questionnaire-9. A range of suicide-related factors was assessed, including sociodemographic characteristics, depression, anxiety, insomnia, pre-existing mental health problems, and COVID-19-related factors.

**Results:**

The prevalence of suicidal ideation was 8.5%, 11.0% and 12.6% at T1, T2, and T3, respectively. Male sex (aOR: 1.35–1.44, *Ps* < 0.001), poor self-perceived mental health (aOR: 2.25–2.81, *Ps* < 0.001), mental diseases (aOR: 1.52–2.09, *P* < 0.001), prior psychological counseling (aOR: 1.23–1.37, *Ps* < 0.01), negative perception of the risk of the COVID-19 epidemic (aOR: 1.14–1.36, *Ps* < 0.001), depressive symptoms (aOR: 2.51–303, *Ps* < 0.001) and anxiety symptoms (aOR: 1.62–101.11, *Ps* < 0.001) were associated with an increased risk of suicidal ideation.

**Conclusion:**

Suicidal ideation appeared to increase during the COVID-19 pandemic remission period among college students in China. Multiple factors, especially mental health problems, are associated with suicidal ideation. Psychosocial interventions should be implemented during and after the COVID-19 pandemic to reduce suicide risk among college students.

## Introduction

The rapid global spread of coronavirus disease 2019 (COVID-19) led to a pandemic, which has been declared by the World Health Organization to be an international crisis [1]. Such a crisis can be emotionally challenging and stressful to all persons affected, especially college students. They bear various pressures, such as academic burdens, financial difficulties and interpersonal relationships [2], and show a stronger stress response to the COVID-19 pandemic [3, 4]. A survey of 2038 Chinese college students conducted by Chi et al. during the COVID-19 outbreak found high levels of mental health problems and distress among college students [5].

In response to the prevention and control measures of the pandemic, the Ministry of Education of the People’s Republic of China announced on January 27, 2020 that the opening of colleges and universities had been postponed and advocated the measures of “classes suspended but learning continues” [6]. Most colleges and universities were using online delivery for the spring semester in 2020 to provide college students with free and open high-quality online courses and teaching resources so that they could carry out online learning, online discussion, Q&A counseling, and online examinations. However, long-term school closures and social distancing during the COVID-19 pandemic may have had a far-reaching impact on the daily lives of college students [7], and the negative psychological effects of social isolation are clear. For instance, the limitations of online learning, such as inefficient learning, lack of eye contact and feedback from teachers, inattention, and difficulty in maintaining academic integrity, are also of concern [8]. Keeping physical distance and losing face-to-face contact with friends may impede the maintenance and development of interpersonal relationships and lead to loneliness, distress, boredom and disappointment. The relatively unfree state and limited activity space may impact and restrict activities that are vital to students’ identity development (e.g., part-time jobs, extracurricular activities), resulting in less physical activity and biorhythm disorders. Staying close to caregivers (almost 24/7) and lacking sufficient personal space in the family may contribute to frequent parent–child conflicts and a surge in family pressure. Moreover, uncertainty about examination and admission arrangements and obstacles to personal development may cause anxiety and concern. The above negative psychological effects of isolation may potentially increase the risk of suicide [9].

The outbreak and prevalence of infectious diseases (such as COVID-19) can lead to an increase in suicide rates [10, 11] and suicidal self-harm behaviors (including suicidal ideas, plans and attempts) [12]. The stress diathesis model [13] posits that stress caused by stressful events (e.g., the COVID-19 pandemic) will cause susceptible people (e.g., college students) to feel anxiety, anger, frustration and despair, affecting their ability to cope with the situation and making them less effective at doing so, which will contribute to suicide. The results of an online survey on the mental health of 1000 Greek university students showed that, in the early stages of the COVID-19 lockdown, students’ suicidal thoughts increased by 63.3% [14]. Additionally, a large repeated cross-sectional study in Poland revealed that, in the first two months of the COVID-19 pandemic in Europe, young adult students (aged 18–24 years) had more symptoms of suicidality than adult students (≥ 25 years) [2]. A cross-sectional online survey in Bangladesh also suggested that suicidal ideation was common among Bangladeshi college students during the COVID-19 pandemic, with an estimated prevalence rate of 12.8% [15]. However, an empirical study on the suicide status of Chinese college students during the COVID-19 pandemic has not been reported. In addition, it is unclear which potential factors of the pandemic may be related to suicidal ideation of college students, and this lack of knowledge may impede appropriate and timely psychological interventions.

Furthermore, widely-reported studies modeling the effect of the COVID-19 pandemic on suicide rates predicted increases ranging from 1 to 145%, largely reflecting variation in underlying assumptions [16, 17]. There have been huge differences in the suicide rates between different countries at different stages of the epidemic, showing inconsistent trends. For example, reports indicated a rise in suicides (Nepal), no rise in suicide rates (USA, Australia, and England), or a decline (Japan, Norway and Peru) in the early months of the pandemic [18–24]. Timely suicide-rate data are crucial. China has gone through three stages in the process of fighting the COVID-19 pandemic, including an outbreak period (where the total confirmed cases in China soared), a remission period (the pandemic in China was basically under control, with zero new confirmed cases in Hubei Province and a decline in the number of newly confirmed cases nationwide) and a normalized prevention and control period (the pandemic situation was generally in a sporadic state, cases originating abroad had been effectively controlled, and pandemic prevention and control work shifted from an emergency state to a normal state). However, to the best of our knowledge, most of the existing studies have had cross-sectional designs. Little is known about suicide status and changing trends among college students since the outbreak of the pandemic, which may hinder educators, managers and policy makers from formulating effective interventions to prevent the adverse effects of social isolation.

**Fig. 1:**
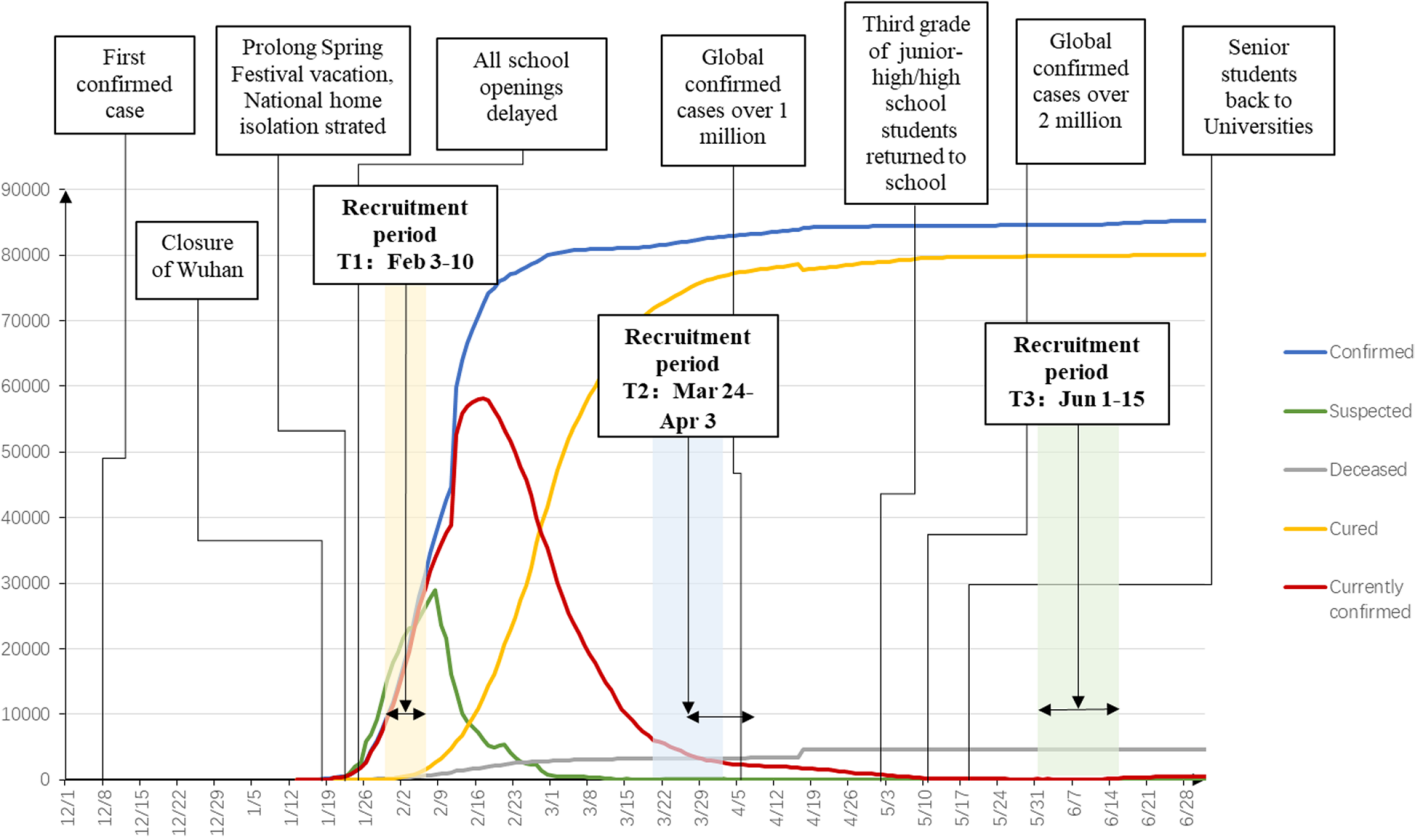
Timeline of the development of the COVID-19 pandemic in China from December 8, 2019, to June 28, 2020

To fill this gap, we conducted a large-scale repeated cross-sectional study of Chinese college students during the outbreak period (T1: February 3 to 10, 2020), remission period (T2: March 24 to April 3, 2020), and normalized prevention and control period (T3: June 1 to 15, 2020) of the COVID-19 pandemic (see Fig. 1), aiming to reveal the prevalence of suicidal ideation and identify its influencing factors among college students in different periods of the COVID-19 pandemic. The goal of the study was help policymakers and school counselors more successfully identify high-risk individuals when planning targeted early interventions.

## Methods

### Participants and procedure

This large-scale repeated cross-sectional survey was conducted in colleges and universities in Guangdong Province, which ranked first behind Hubei Province in the number of confirmed cases at the outbreak stage of the pandemic in China. In total, 22 colleges and universities, which included 11 undergraduate colleges and technical colleges, were selected to participate in the current survey, which was conducted from February 3 to 10 (T1), March 24 to April 3 (T2) and June 1 to 15 (T3) in 2020. We prepared a normative notice applicable to these 22 schools, including the online survey’s purpose, significance, deadline and mode of participation. The director of each school’s psychological counseling center or full-time psychological teacher was the responsible party, and they were responsible for sending the above notice to each student via WeChat or QQ and communicating closely with us about any problems or difficulties encountered. Participants could use WeChat to access the survey and answer the online questionnaire by scanning the two-dimensional barcode or clicking on the relevant link within the survey period. They were asked to carefully read the instructions about the purpose and method of filling out the questionnaire. All of the participants volunteered to participate in the study and were allowed to withdraw at any time. We obtained the participants’ online informed consent before formal implementation of the survey. Confidentiality of data and participants’ personal information was ensured. After completing the questionnaire, participants could take part in the lottery, and the prizes were generally discount coupons.

The contents of the questionnaire consisted of demographic data, factors related to the COVID-19 pandemic, psychosocial factors, and mental health status (including depressive, anxiety, and insomnia symptoms, and suicidal ideation). The effective sample sizes of T1, T2, and T3 were 164,101, 148,384, and 159,187, respectively, and the effective rates were 88.3%, 95.4%, and 95.7%, respectively. It is worth noting that since the three samples of this study were independent, some students may have participated in the survey for more than one wave, but we did not track these parts. This study was approved by the appropriate institutional research and ethics committee.

### Measurement

#### Demographics and COVID-19 related information

The participants provided demographic information including age, sex (male or female), grade (freshman, sophomore, junior, senior, or graduate), family residence (rural, town, small city, or major city), psychiatric history (yes or no), physical health problems (yes or no), counseling history (yes or no), self-perceived physical and mental health (good, average or poor), psychological assistance for COVID-19 (yes or no), beliefs toward the COVID-19 pandemic, and social media exposure to COVID-19 (< 1 h/day, 1–2 h/day or > 2 h/day).

#### Suicidal ideation

Suicidal ideation was evaluated by item 9 of the Patient Health Questionnaire (PHQ) (referred to hereafter as the “PHQ-9 suicide item”), which was used to evaluate the frequency of passive thoughts of death or self-injury within the last two weeks. The PHQ-9 suicide item offers a score of 0 to 3 to answer the following question: "How often have you been bothered by thoughts that you would be better off dead or of hurting yourself in some way?" Possible answers include "not at all" (0), "a few days" (1), "more than half of the days" (2), and "almost every day" (3). The PHQ-9 suicide item has been widely used as a single measure to assess suicidal ideation in previous studies [25]. Any score on the PHQ-9 suicide item greater than 0 indicates probable suicidal ideation [26, 27].

#### Depressive symptoms

The 9-item Patient Health Questionnaire (PHQ-9) is a self-report scale consisting of 9 items assessing an individual’s depressive symptoms over the previous 2 weeks [28]. The PHQ-9 is rated on a 4-point Likert scale, and the total score ranges from 0 to 27, with higher scores indicating more serious depressive symptoms. In the current study, we used the score of the first eight items of the PHQ-9 to evaluate depressive symptoms (referred to hereafter as the “PHQ-8”). Cronbach’s α for the PHQ-8 in the current study was 0.88 at T1, 0.91 at T2, and 0.92 at T3.

#### Anxiety symptoms

The Chinese version of the Generalized Anxiety Disorder Scale (GAD-7) is a 7-item self-report scale that is widely used to evaluate anxiety symptoms [29]. The participants were asked to report their symptoms of anxiety over the previous 2 weeks on a Likert scale from 0 (not at all) to 3 (nearly every day), for a total score ranging from 0 to 21. A higher score indicated a higher degree of anxiety symptoms. Cronbach’s α for the GAD-7 in the current study was 0.92 at T1, 0.93 at T2, and 0.94 at T3.

#### Insomnia symptoms

The Youth Self-Rating Insomnia Scale (YSIS) is a brief, reliable and valid tool to assess insomnia symptoms, perceived sleep quality and insufficiency, and impaired daytime functioning among Chinese youth [30]. The YSIS consists of 8 items, each of which is rated on a 5-point scale. Summing the scores of the 8 items yields the total YSIS score, ranging from 8 to 40. A higher total score on the YSIS indicates greater insomnia severity during the previous month. Cronbach’s α for the YSIS in the current study was 0.89 at T1, 0.90 at T2, and 0.90 at T3.

### Statistical analyses

Data were analyzed with SPSS Version 25.0. Descriptive statistics, including frequencies and central tendencies, were calculated to characterize the sample’s demographic profile. The chi-square test was used to test the difference between the prevalence of suicidal ideation from T1 to T3. Binary and multiple logistic regression analyses were performed to explore the potential factors influencing suicidal ideation during the COVID-19 pandemic. The three mental health symptoms (depression, anxiety, and insomnia) were divided into four groups using quartiles, indicating the position of the score in the sample. The first quartile was 0–25%, the second was 25%-50%, the third was 50%-75%, and the fourth was 75%-100%. Odds ratios (ORs) and 95% confidence intervals (95% CIs) were obtained from the logistic regression models. *P* < 0.05 was considered statistically significant.

## Results

### Descriptive characteristics

The descriptive characteristics of the participants are reported in Table 1. In the three-wave survey, the average age of the college students was approximately 20 years old. Freshmen accounted for the largest proportion (> 30%). The number of females was approximately three times that of males, with T1 females accounting for 63.16%, T2 females accounting for 62.6%, and T3 females accounting for 62.14%. Detailed sample demographics and health behavior at the three time points are shown in Table 1.

**Table 1:** Descriptive characteristics of the sampled college students

Variables	T1 N (%)	T2 N (%)	T3 N (%)
**Sex**			
Male	60,456(36.84%)	55,484(37.40%)	60,270(37.86%)
Female	103,645(63.16%)	92,859(62.60%)	98,917(62.14%)
**Grade**			
Freshman year	51,963(31.67%)	47,665(32.10%)	50,452(31.69%)
Sophomore year	46,110(28.10%)	41,685(28.10%)	45,600(28.65%)
Junior year	38,656(23.56%)	33,522(22.60%)	35,107(22.05%)
Senior year	20,740(12.64%)	17,008(11.47%)	19,091(11.99%)
Graduate students	6632(4.04%)	8463(5.71%)	8937(5.61%)
**Residence**			
Rural area	-	59,587(40.20%)	59,209(37.19%)
Town	-	41,782(28.20%)	45,084(28.32%)
Small and medium-sized cities	-	29,408(19.80%)	33,158(20.83%)
Major cities	-	17,566(11.80%)	21,736(13.65%)
**Major physical health problem**			
Yes	822(0.50%)	942(0.64%)	531(0.33%)
No	163,279(99.50%)	147,401(99.36%)	158,656(99.67%)
**Self-perceived physical health**			
Good	155,257(94.6%)	141,501(95.4%)	151,965(95.5%)
Average	8632(5.3%)	6651(4.5%)	6834(4.3%)
Poor	212(0.1%)	191(0.1%)	388(0.2%)
**Mental illness**			
Yes	1432(0.87%)	1359(0.92%)	1287(0.81%)
No	162,669(99.13%)	146,984(99.08%)	157,900(99.19%)
**Self-perceived mental health**			
Good	149,251(91.0%)	131,097(88.4%)	139,526(87.6%)
Average	14,004(8.5%)	16,136(10.9%)	17,579(11.0%)
Poor	846(0.5%)	1110(0.7%)	2082(1.4%)
**Ever received counseling from a professional**			
Yes	7845(4.78%)	7402(4.99%)	7953(5.00%)
No	156,256(95.22%)	140,941(95.01%)	151,234(95.00%)
**Psychological assistance since the outbreak**			
Yes	760(0.46%)	811(0.55%)	1047(0.66%)
No	163,341(99.54%)	147,532(99.45%)	158,140(99.34%)
**COVID-19 prevention beliefs**			
Yes	157,846(96.19%)	145,211(97.89%)	155,518(97.70%)
No	6255(3.81%)	3132(2.11%)	3669(2.30%)
**COVID-19 curative beliefs**			
Yes	158,790(96.76%)	144,900(97.68%)	154,280(96.92%)
No	5311(3.24%)	3443(2.32%)	4907(3.08%)
**Implement COVID-19 preventive actions**			
Yes	157,609(96.04%)	145,118(97.83%)	155,803(97.87%)
No	6492(3.96%)	3225(2.17%)	3384(2.13%)
**Social media exposure**			
< 1 h/Day	62,786(38.3%)	92,239(62.2%)	116,090(72.9%)
1–2 h/Day	75,073(45.7%)	47,520(32.0%)	37,791(23.8%)
> 2 h/Day	26,242(16.0%)	8584(5.8%)	5306(3.3%)
**YSIS**			
Q1	47,661(29.0%)	45,344(30.6%)	40,858(25.7%)
Q2	36,085(22.0%)	36,664(24.7%)	43,876(27.6%)
Q3	43,401(26.4%)	31,069(20.9%)	36,205(22.7%)
Q4	36,954(22.5%)	35,266(23.8%)	38,248(24.0%)
**PHQ-8**			
Q1	47,252(28.8%)	41,350(27.9%)	37,321(23.4%)
Q2	46,539(28.4%)	34,626(23.3%)	42,305(26.6%)
Q3	34,395(21.0%)	41,448(27.9%)	60,485(38.0%)
Q4	35,915(21.9%)	30,919(20.8%)	19,076(12.0%)
**GAD-7**			
Q1	0	73,566(49.6%)	70,818(44.5%)
Q2	83,942(51.2%)	14,907(10.0%)	13,524(8.5%)
Q3	40,592(24.7%)	23,848(16.1%)	41,908(26.3%)
Q4	39,567(24.1%)	36,022(24.3%)	32,937(20.7%)

Variables including family residence were not measured at T1 and are represented by “- “. YSIS represents the total score of the Youth Self-Rating Insomnia Scale, PHQ-8 represents the total score of the first eight items of the 9-item Patient Health Questionnaire depressive symptoms, and GAD-7 represents the total score of the Generalized Anxiety Disorder Scale. Q1 represents the first quartile, Q2 represents the second quartile, Q3 represents the third quartile, and Q4 represents the fourth quartile

### Prevalence of suicidal ideation at T1-T3

**Fig. 2:**
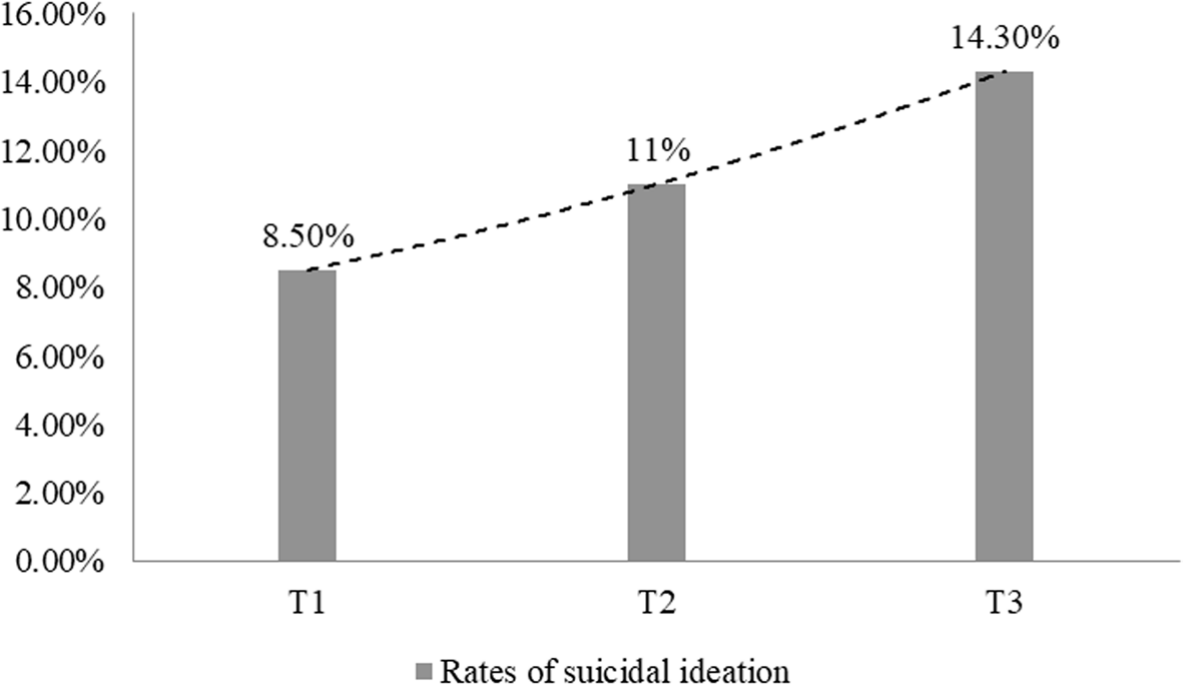
Bar graph of the prevalence of suicidal ideation

As shown in Fig. 2, the prevalence of suicidal ideation at T1, T2 and T3 was 8.5% (95% CI: [8.3, 8.6]), 11.0% (95% CI: [10.8, 11.1]) and 14.3% (95% CI: [14.2, 14.5]), respectively. The χ^2^ test between the groups was significant, χ^2^ = 2795.35, *P* < 0.001, showing an overall upward trend.

### Logistic regression analysis of factors associated with suicidal ideation

The binary and multiple logistic regression analysis results for the risk factors for suicidal ideation are shown in Table 2. Binary and multiple logistic regression was used to calculate the independent odds ratio (Crude OR, cOR) and adjusted odds ratio (Adj OR, aOR) of the risk factors for suicidal ideation, which were used to reflect the OR of each factor individually and jointly when predicting suicidal ideation. Factors influencing suicidal ideation include sex; grade; self-perceived physical health; self-perceived mental health; major physical health problem; mental illness; psychological counseling experience; psychological assistance for the COVID-19 pandemic; daily time consuming epidemic information; awareness of the COVID-19 pandemic; and insomnia, depression, and anxiety.

**Table 2:** Factors associated with suicidal ideation at T1, T2, and T3

Variables	Category	T1				T2				T3			
		**Crude OR**	**95% Cl**	**Adj OR**	**95% Cl**	**Crude OR**	**95% Cl**	**Adj OR**	**95% Cl**	**Crude OR**	**95% Cl**	**Adj OR**	**95% Cl**
**Age**		0.98^***^	(0.97,0.99)	0.97^***^	(0.95,0.98)	0.99^*^	(0.98,1.00)	1.00	(0.98,1.02)	0.96^***^	(0.96,0.97)	0.97^**^	(0.96,0.99)
**Sex**	**Female**	-		-	-	-		-	-	-		-	-
	**Male**	1.06^**^	(1.03,1.10)	1.44^***^	(1.38,1.50)	1.18^**^	(1.14,1.22)	1.42^***^	(1.37,1.48)	1.15^***^	(1.12,1.19)	1.35^***^	(1.30,1.40)
**Grade**	**Freshman**	-	-	-	-	-	-	-	-	-	-	-	-
	**Sophomore**	0.99^*^	(1.04,1.14)	1.10^***^	(1.04,1.17)	1.00	(1.06,1.15)	1.05	(0.99,1.11)	0.97	(1.05,1.13)	1.06^**^	(1.01,1.11)
	**Junior**	1.11^***^	(1.06,1.17)	1.12^***^	(1.05,1.20)	1.09^***^	(1.04,1.14)	0.94	(0.87,1.00)	1.17^***^	(1.12,1.21)	1.04	(0.98,1.10)
	**Senior**	1.15^***^	(1.09,1.21)	1.06	(0.98,1.14)	1.10^***^	(1.05,1.17)	0.72^***^	(0.65,0.78)	0.80^***^	(0.76,0.84)	0.80^***^	(0.74,0.87)
	**Graduate**	0.61^***^	(0.55,0.69)	0.60^***^	(0.51,0.71)	0.68^***^	(0.62,0.74)	0.49^***^	(0.42,0.56)	0.66^***^	(0.62,0.72)	0.62^***^	(0.54,0.70)
**Self-perceived physical health**	**Good**	-	-	-	-	-	-	-	-	-	-	-	-
	**Average**	3.29^***^	(3.11,3.47)	1.05	(0.98,1.13)	3.41^***^	(3.22,3.61)	1.07	(0.99,1.15)	3.61^***^	(3.42,3.80)	1.06	(0.99,1.14)
	**Poor**	5.53^***^	(4.13,7.39)	1.27	(0.86,1.85)	7.91^***^	(5.95,0.00)	1.20	(0.84,1.72)	5.52^***^	(4.47,0.00)	0.97	(0.75,1.24)
**Self-perceived mental health**	**Good**	-		-	-	-		-	-	-		-	-
	**Average**	4.85^***^	(4.64,5.06)	1.44^***^	(1.36,1.52)	4.30^***^	(4.14,4.48)	1.25^***^	(1.18,1.31)	4.20^***^	(4.05,4.36)	1.35^***^	(1.29,1.42)
	**Poor**	15.73^***^	(13.72,18.03)	2.81^***^	(2.39,3.29)	15.13^***^	(13.41,17.08)	2.32^***^	(2.01,2.67)	12.16^***^	(11.09,13.34)	2.25^***^	(2.02,2.51)
**Major physical health problem**	**No**	-		-	-	-		-	-	-		-	-
	**Yes**	1.73***	(1.42,2.11)	1.33*	(1.03,1.70)	1.90***	(1.61,2.24)	1.37**	(1.10,1.71)	1.83***	(1.49,2.23)	0.96	(0.74,1.26)
**Mental illness**	**No**	-	-	-	-	-	-	-	-	-	-	-	-
	**Yes**	4.65***	(4.15,5.22)	1.65***	(1.41,1.92)	3.62***	(3.22,4.07)	1.52***	(1.30,1.79)	4.37***	(3.91,4.89)	2.09***	(1.78,2.46)
**Ever received counseling from a professional**	**No**	-	-	-	-	-	-	-	-	-	-	-	-
	**Yes**	2.74***	(2.59,2.91)	1.37***	(1.27,1.48)	2.32***	(2.19,2.46)	1.23***	(1.14,1.33)	2.41***	(2.29,2.53)	1.24***	(1.16,1.33)
**Psychological assistance since the outbreak**	**No**	-	-	-	-	-	-	-	-	-	-	-	-
	**Yes**	2.20***	(1.82,2.66)	1.04	(0.83,1.31)	2.81***	(2.39,3.29)	1.14	(0.94,1.40)	3.13***	(2.75,3.55)	1.12	(0.95,1.33)
**Social media exposure**	** < 1 h/Day**	-	-	-	-	-	-	-	-	-	-	-	-
	**1–2 h/Day**	0.84^***^	(0.81,0.87)	0.82^***^	(0.78,0.86)	1.11^***^	(1.07,1.15)	0.98	(0.94,1.03)	1.13^***^	(1.09,1.17)	1.12^***^	(1.07,1.17)
	** > 2 h/Day**	1.06^**^	(1.01,1.12)	0.92^***^	(0.87,0.98)	1.46^***^	(1.37,1.56)	1.20^***^	(1.10,1.30)	1.42^***^	(1.31,1.53)	1.36^***^	(1.24,1.50)
**Covid-19 prevention beliefs**	**Yes**	-	-	-	-	-	-	-	-	-	-	-	-
	**No**	2.24^***^	(2.09,2.40)	1.19^***^	(1.09,1.30)	2.31^***^	(2.12,2.52)	1.32^***^	(1.17,1.48)	2.29^***^	(2.12,2.48)	1.34^***^	(1.21,1.49)
**Implement Covid-19 preventive actions**	**Yes**	-	-	-	-	-	-	-	-	-	-	-	-
	**No**	2.22^***^	(2.07,2.37)	1.30^***^	(1.19,1.41)	2.37^***^	(2.18,2.58)	1.36^***^	(1.21,1.52)	2.25^***^	(2.08,2.44)	1.35^***^	(1.22,1.50)
**Covid-19 curative beliefs**	**Yes**	-	-	-	-	-	-	-	-	-	-	-	-
	**No**	2.19^***^	(2.03,2.36)	1.20^***^	(1.09,1.32)	2.18^***^	(2.00,2.37)	1.14^**^	(1.03,1.27)	2.08^***^	(1.95,2.23)	1.22^***^	(1.12,1.33)
**YSIS**	**Q1**	-	-	-	-	-	-	-	-	-	-	-	-
	**Q2**	2.17^***^	(2.00,2.35)	0.96	(0.88,1.05)	2.33^***^	(2.17,2.51)	0.76^***^	(0.69,0.83)	2.40^***^	(2.23,2.59)	0.79^***^	(0.73,0.86)
	**Q3**	4.46^***^	(4.15,4.79)	0.87^***^	(0.80,0.94)	6.35^***^	(5.93,6.79)	0.80^***^	(0.73,0.87)	7.65^***^	(7.16,8.18)	1.00	(0.92,1.08)
	**Q4**	12.79^***^	(11.94,13.69)	0.92^*^	(0.85,1.00)	13.43^***^	(12.59,14.32)	0.67^***^	(0.62,0.73)	13.40^***^	(12.56,14.30)	0.81^***^	(0.74,0.88)
**PHQ-8**	**Q1**	-	-	-	-	-	-	-	-	-	-	-	-
	**Q2**	5.18^***^	(4.32,6.21)	3.44^***^	(2.85,4.14)	5.02^***^	(4.02,6.26)	4.18^***^	(3.34,5.24)	3.60^***^	(2.89,4.48)	2.51^***^	(2.03,3.11)
	**Q3**	19.92^***^	(16.78,23.64)	7.28^***^	(6.06,8.75)	30.77^***^	(25.14,37.65)	12.36^***^	(9.96,15.35)	23.02^***^	(18.97,27.94)	11.57^***^	(9.42,14.22)
	**Q4**	149.88 ^***^	(126.86,177.09)	29.85 ^***^	(24.86,35.84)	303.00 ^***^	(248.20,369.90)	59.90 ^***^	(48.17,74.49)	254.56 ^***^	(210.35,308.06)	10.63 ^***^	(8.61,13.12)
**GAD-7**	**Q1**	-	-	-	-	-	-	-	-	-	-	-	-
	**Q2**	-	-	-	-	3.27^***^	(2.89,3.69)	1.62^***^	(1.43,1.84)	5.31^***^	(4.66,6.06)	2.72^***^	(2.38,3.11)
	**Q3**	5.46^***^	(5.01,5.95)	2.43^***^	(2.22,2.66)	7.24^***^	(6.61,7.94)	2.37^***^	(2.14,2.62)	10.16^***^	(9.15,11.28)	5.28^***^	(4.75,5.87)
	**Q4**	44.49 ^***^	(41.26,47.97)	8.08 ^***^	(7.41,8.82)	64.69 ^***^	(59.83,69.96)	9.32 ^***^	(8.49,10.22)	101.11 ^***^	(92.05,111.06)	44.82 ^***^	(40.33,49.80)

^***^P < 0.001, ^**^P < 0.01, ^*^P < 0.05. YSIS represents the total score of the Youth Self-Rating Insomnia Scale, PHQ-8 represents the total score of the first eight items of the 9-item Patient Health Questionnaire depressive symptoms, and GAD-7 represents the total score of the Generalized Anxiety Disorder Scale. Q1 represents the first quartile, Q2 represents the second quartile, Q3 represents the third quartile, and Q4 represents the fourth quartile

All the influencing factors could independently predict suicidal ideation, but there were differences when they were jointly predicted. For instance, being senior at T2 (aOR = 0.72, 95% CI: (0.65,0.78), *P* < 0.001) and T3 (aOR = 0.8, 95% CI: (0.74,0.87), *P* < 0.001) was a protective factor for suicidal ideation when predicting suicidal ideation jointly. Self-perceived physical health and psychological assistance for the COVID-19 pandemic could independently predict suicidal ideation, but the aOR was not significant from T1 to T3 when predicting suicidal ideation jointly (*Ps* > 0.05). Consuming pandemic information for more than 2 h daily was a risk factor for suicidal ideation when it was predicted separately (T1: cOR = 1.06, 95% CI: (1.01,1.12), *P* < 0.01; T2: cOR = 1.46, 95% CI: (1.37,1.56), *P* < 0.001; T3: cOR = 1.42, 95% CI: (1.31,1.53), *P* < 0.001). However, when jointly predicted, this variable became a protective factor at T1 (aOR = 0.92, 95% CI: [0.87,0.98], *P* < 0.001) and remained a risk factor at T2 (aOR = 1.20, 95% CI: (1.10,1.30), *P* < 0.001) and T3 (aOR = 1.36, 95% CI: (1.24,1.50), *P* < 0.001). Depression and anxiety were positively correlated with suicidal ideation when predicted individually and jointly (*Ps* < 0.001), while insomnia symptoms were positively correlated when predicting suicidal ideation alone (*Ps* < 0.001), but the significant aOR of joint prediction was less than 1.

## Discussion

In one of the first large-scale, 3-wave cross-sectional studies of suicidal ideation among Chinese home-quarantined college students during the COVID-19 pandemic, our findings reveal a clear upward trend in the prevalence of suicidal ideation among college students during the outbreak, remission and normalized prevention and control periods, which is consistent with the research results of Debowska [2] and Connor [31]. The high and rising suicidal ideation in this study confirms the profound negative impact of the COVID-19 pandemic on college students. The secondary consequences brought about by the pandemic’s continued impact, including restrictions on movement outdoors, obstacles in living arrangements, limited interpersonal communication, postponement or cancellation of academic activities and large-scale examinations related to personal development, make college students feel distressed and worried. They can easily experience frustration when resolving various key life changes (e.g., education, professional development, social and romantic relationships). This frustration is sometimes so overwhelming that they may consider coping with their fear, doom and despair by self-harm or suicide. As Shneidman’s psychache theory holds [32], suicide is the only way to solve the problem caused by psychological pain. Once psychological pain exceeds the scope of what a person can bear, suicide will occur. Although the pandemic has been brought under control in China, the prevalence of suicidal ideation is still on the rise, which suggests that we need to remain vigilant about suicide among college students, continue to monitor and track suicidal ideation and identify appropriate response and intervention strategies.

In this study, male sex, poor self-perceived mental health, history of mental illness, counseling experience, depression, anxiety and negative pandemic perception (the belief that the COVID-19 pandemic is unpreventable and untreatable) were risk factors for suicidal ideation, which was consistent with previous studies. A cross-sectional survey of 560,000 Chinese adults in China [25] showed that among college students, the prevalence of suicidal ideation in males (19.1%) was higher than that in females (11.9%), possibly because males were vulnerable to depression, insomnia, and acute stress during the COVID-19 pandemic [33]. Self-perceived mental health is relatively stable between 23 and 33 years old and is related to psychological disturbance. Both self-perceived mental health and counseling experiences reflect the level of individual perception of psychological problems and reflect the seriousness of the psychological problems that individuals are experiencing. Previous research has suggested that individuals who seek counseling are more likely to experience symptoms of fear, trauma, and depression than individuals who do not seek counseling [34]. Chinese college students tend to seek help from outsiders only when they encounter serious psychological problems [35]. That is an avoidant coping strategy that is positively related to Chinese young adults’ psychological symptoms [36] and is generally associated with greater psychological distress [37]. Unstable mental health conditions can easily lead to individual suicide, which is more pronounced in individuals with pre-existing mental illness because they tend to be unable to cope with stressful environments [38], such as the COVID-19 pandemic. They may experience more severe symptoms and new mental health problems, especially depression, anxiety, and posttraumatic stress disorder, which are both associated with an increased risk of suicide. In addition, there was a significant correlation between the appearance of stress symptoms and people’s cognition of COVID-19. Studies have confirmed that people who believe that the epidemic is "unpreventable and incurable" have higher stress levels and a higher prevalence of symptoms [39]. Negative perceptions may make the pandemic look more catastrophic and dangerous, which in turn makes students feel helpless, causing them to underestimate their coping abilities and experience more psychological stress.

Inconsistent with previous studies, the results of the current study indicated that when the pandemic moved into remission and became normalized in the prevention and control period, college seniors had less suicidal ideation than students in the other years. Related studies found that uncertainty in the early stages of the pandemic increased anxiety about graduating, getting a job or going on to further education and thus increased the risk of developing mental health problems [40]. However, the Ministry of Education of the People’s Republic of China has formulated timely psychological counseling and employment policies [41], such as encouraging enterprises to provide jobs, organizing online job fairs, broadening employment channels, encouraging independent entrepreneurship, and strengthening psychological counseling for employment, effectively helping these students obtain employment and providing compensatory opportunities for further studies, which somewhat alleviates the psychological pressure on college seniors.

In this study, spending more than 2 h daily consuming information on the pandemic was a risk factor for suicidal ideation in T2 and T3, which is consistent with the results of Li et al. [42]. The longer one reads about COVID-19 every day, the more likely one is to have negative perceptions. Although large-scale pandemic media broadcasts can provide people with information to better understand the epidemic situation and nature of the disease, the information published and spread by many networks and media channels contains erroneous content and rumors, which can lead to (erroneous) information overload and subsequently to mental health problems [43]. In addition, as people pay attention to updated information on new cases and deaths every day, awareness of the seriousness of COVID-19 has grown, leading to a significant increase in panic, psychological stress, and suicidal ideation. Different from previous studies, the pandemic focus time of more than 2 h in this study was a protective factor against suicidal ideation during the outbreak period, which may be due to the lack of understanding and awareness of COVID-19 at the beginning of the pandemic. In the early days of a pandemic, people need to obtain information to understand the situation. With an in-depth understanding of the nature of the pandemic, the sense of control will increase, and the experience of internal control will be enhanced, which should create a buffer against psychological stress and thereby reduce suicidal ideation [44].

During the COVID-19 pandemic, depression and anxiety were risk factors for suicidal ideation in college students, which is in line with previous findings [2]. Any major outbreak will harm individuals and society, causing depression and anxiety, which are independent predictors of suicide [45, 46]. College students are young, lack social experience, experience fluctuating emotions, and lack coping experience and related knowledge. In this study, their stress response was more prominent, and the depression score and the proportion of moderate to severe depression increased significantly [47]. The COVID-19 outbreak has caused college students to face many life changes, which are stressful [48] and may lead to depression and suicidal thoughts while they are trying to cope and deal with stress. Wang and Zhao [49] evaluated the anxiety status of 3611 Chinese college students in the early stage of the outbreak and found that they showed high anxiety about COVID-19. Some studies conducted in China during SARS and H1NI also showed that college students felt obvious anxiety and pressure [50, 51]. The anxiety of college students during COVID-19 was higher than that during the outbreaks of SARS and H1N1 influenza. This may be due to the prolonged home isolation of the pandemic, which exposes college students to various stressors and easily causes chronic distress and emotional disorders, which are related to suicidal thoughts [52].

In addition, this study found that insomnia was a risk factor for suicidal ideation when predicted alone, while when jointly predicted, insomnia became a protective factor for suicidal ideation. Sleep problems are highly likely to occur or worsen during outbreaks [53]. On the one hand, existing studies have shown that the prevalence of insomnia among college students during the pandemic period of home study is high [54]. Persistent insomnia increases the risk of depression and suicide [55]. Previous studies have found that fatigue, social problem-solving ability, and despair partly explain the relationship between insomnia and suicide, which is consistent with the dual-process theories and social problem-solving model [56]. Fatigue caused by insomnia may increase the difficulty of dealing with and managing daily affairs, making people more vulnerable to negative emotions and perceived self-exhaustion, causing feelings of despair and suicidal ideation. On the other hand, in the joint prediction, the protective factor of insomnia turning into suicidal ideation may be related to a number of the variables included in the prediction model. Because there were many variables included in the equation for joint prediction, the effect of insomnia was weakened or interacted with other factors. Other factors (such as anxiety and depression) may explain this relationship to a certain extent; thus, the above results were produced.

The limitations of this paper are as follows: (1) Due to the voluntariness of participation in the research, the impact of the COVID-19 epidemic on college students’ suicidal ideation may have been over or underestimated. We cannot know the prevalence of suicidal ideation of college students who did not participate in the survey. (2) The cross-sectional design limited the explanation of causality. The reasons for the increasing prevalence of suicidal ideation among college students and the causal relationship between multiple risk factors, such as quarantine and suicidal ideation still need to be explained by longitudinal data. (3) The PHQ-9 suicide item, a single response item, may have biased the results, and it was not able to explore other domains of suicidality, such as suicidal plans and attempts. Subsequent studies can use the Columbia-Suicide Severity Rating Scale (C-SSRS) [57] or other indicators to comprehensively assess the severity of suicidal ideation.

## Conclusion

In conclusion, this study first described the prevalence of suicidal ideation among Chinese college students during the three key windows of the COVID-19 pandemic. The COVID-19 pandemic has had a long-term and far-reaching negative impact on the mental health of Chinese college students in home isolation, leading to a continuous increase in suicidal ideation. These findings contribute to a better understanding of the prevalence of suicidal ideation among college students since the outbreak and remind us to pay close attention to the mental health of college students after the pandemic. We suggest continuous monitoring of the psychological status of college students, encouraging them to conduct self-monitoring and reporting, timely screening and identification of individuals at high risk of suicide, and providing targeted psychological intervention services.

## Data Availability

The datasets generated and/or analyzed during the current study contain clinical data and are not publicly available due to the protection of participants’ rights to privacy and data protection but are available from the corresponding author on reasonable request.

## References

[1] Zhou F, Yu T, Du R, Fan G, Liu Y, Liu Z, Cao B (2020). Clinical course and risk factors for mortality of adult inpatients with COVID-19 in Wuhan, China: a retrospective cohort study. Lancet.

[2] Debowska A, Horeczy B, Boduszek D, Dolinski D (2020). A repeated cross-sectional survey assessing university students’ stress, depression, anxiety, and suicidality in the early stages of the COVID-19 pandemic in Poland. Psychol Med.

[3] Xu W, Jiang HL, Zhang Y, An YY (2019). Responsiveness and predictors of red cross rescue workers’ psychological trauma. Chin J Clin Psychol.

[4] Wang C, Pan R, Wan X, Tan Y, Xu L, Ho CS, Ho RC (2020). Immediate psychological responses and associated factors during the initial stage of the 2019 coronavirus disease (COVID-19) epidemic among the general population in China. Int J Environ Res Public Health.

[5] Chi X, Becker B, Yu Q, Willeit P, Jiao C, Huang L, Hossain MM, Grabovac I, Yeung A, Lin J, Veronese N, Wang J, Zhou X, Doig SR, Liu X, Carvalho AF, Yang L, Xiao T, Zou L, Fusar-Poli P, Solmi M (2020). Prevalence and psychosocial correlates of mental health outcomes among Chinese college students during the coronavirus disease (COVID-19) pandemic. Front Psychiatry.

[6] The ministry of education of the people’s republic of China (2020). Notice of the ministry of education on the postponement of the 2020 spring semester.

[7] Bian HM, Pan T, Zhao M (2020). Emotional analysis of college students in the early stage of COVID-19 epidemic. Chin J School Health.

[8] Mukhtar K, Javed K, Arooj M, Sethi A (2020). Advantages, limitations and recommendations for online learning during COVID-19 pandemic era. Pak J Med Sci.

[9] Thakur V, Jain A (2020). COVID 2019-suicides: a global psychological pandemic. Brain Behav Immun.

[10] Sher L (2020). The impact of the COVID-19 pandemic on suicide rates. QJM.

[11] Sher L. An infectious disease epidemic and resilience to suicide. Aust N Z J Psychiatry. 2020b; 486742092447. 10.1177/0004867420924475 10.1177/0004867420924475 32363912

[12] Chan SMS, Chiu FKH, Lam CWL, Leung PYV, Conwell Y (2006). Elderly suicide and the 2003 SARS epidemic in Hong Kong. Int J Geriatr Psychiatry.

[13] Wasserman D (2003). Suicide: an unnecessary death.

[14] Kaparounaki CK, Patsali ME, Mousa D-PV, Papadopoulou EVK, Papadopoulou KKK, Fountoulakis KN (2020). University students’ mental health amidst the COVID-19 quarantine in Greece. Psychiatry Res.

[15] Tasnim R, Islam MS, Sujan MSH, Sikder MT, Potenza MN (2020). Suicidal ideation among Bangladeshi university students early during the COVID-19 pandemic: prevalence estimates and correlates. Child Youth Serv Rev.

[16] John A , Okolie C, Eyles E, Webb RT, Gunnell D. The impact of the covid-19 pandemic on self-harm and suicidal behaviour: a living systematic review [version 1; peer review: awaiting peer review]. F1000Res. 2020;9. 10.12688/f1000research.25522.1 10.12688/f1000research.25522.1 PMC7871358 33604025

[17] John A, Pirkis J, Gunnell D, Appleby L, Morrissey J (2020). Trends in suicide during the covid-19 pandemic. BMJ.

[18] Ueda M, Nordström R, Matsubayashi T. Suicide and mental health during the COVID-19 pandemic in Japan. J Public Health. 2020;fdab113. 10.1093/pubmed/fdab113 10.1093/pubmed/fdab113 PMC8083330 33855451

[19] Calderon-Anyosa R, Kaufman J (2020). Impact of COVID-19 lockdown policy on homicide, suicide, and motor vehicle deaths in Peru. Prev Med.

[20] Faust J, Shah S, Du C, Li S, Lin Z, Krumholz H. Suicide deaths during the stay-at-home advisory in Massachusetts. medRxiv 2020. [Preprint.] 10.1101/2020.10.20.20215343

[21] Pokhrel S, Sedhai YR, Atreya A. An increase in suicides amidst the coronavirus disease 2019 pandemic in Nepal. Med Sci Law. 2020;25802420966501. 10.1177/0025802420966501 Pmid: 33036544 10.1177/0025802420966501 33036544

[22] Coroners Court of Victoria (2020). Coroners Court monthly suicide data report. Report 2.

[23] Appleby L, Kapur N, Turnbull P, Nicola Richards and the National Confidential Inquiry team. National Confidential Inquiry into Suicide and Safety in Mental Health. Suicide in England since the COVID-19 pandemic- early figures from real-time surveillance. 2020. http://documents.manchester.ac.uk/display.aspx?DocID=51861

[24] Qin P, Mehlum L (2020). National observation of death by suicide in the first 3 months under COVID-19 pandemic. Acta Psychiatr Scand.

[25] Shi L, Que J, Lu Z, Gong Y, Liu L, Wang Y (2021). Prevalence and correlates of suicidal ideation among the general population in china during the covid-19 pandemic. Eur Psychiatry.

[26] Kroenke K, Spitzer RL, Williams JBW (2001). The PHQ-9: validity of a brief depression severity measure. J Gen Intern Med.

[27] Schulberg HC, Lee PW, Bruce ML, Raue PJ, Lefever JJ, Williams JW, Nutting PA (2005). Suicidal ideation and risk levels among primary care patients with uncomplicated depression. Ann Fam Med.

[28] Zhang YL, Liang W, Chen ZM, Zhang HM, Zhang JH, Weng XQ (2013). V alidity and reliability of patient health questionnaire-9 and patient health questionnaire-2 to screen for depression among college students in China. Asia Pac Psychiatry.

[29] Spitzer RL, Kroenke K, Williams JB, Lowe B (2006). A brief measure for assessing generalized anxiety disorder: the GAD-7. Arch Intern Med.

[30] Liu X, Yang Y, Liu Z-Z, Luo Y, Fan F, Jia C-X (2019). Psychometric properties of Youth Self-Rating Insomnia Scale (YSIS) in Chinese adolescents. Sleep Biol Rhythms.

[31] O'Connor RC, Wetherall K, Cleare S, McClelland H, Robb KA (2020). Mental health and wellbeing during the covid-19 pandemic: longitudinal analyses of adults in the uk covid-19 mental health & wellbeing study. Br J Psychiatry.

[32] Shneidman ES (1993). Suicide as psychache. J Nerv Ment Dis.

[33] Shi L, Lu ZA, Que JY, Huang XL, Liu L, Ran MS (2020). Prevalence of and risk factors associated with mental health symptoms among the general population in China during the coronavirus disease 2019 pandemic. JAMA Netw Open.

[34] Liang SW, Chen RN, Liu LL, Li XG, Chen JB, Tang SY (2020). The psychological impact of the covid-19 epidemic on guangdong college students: the difference between seeking and not seeking psychological help. Front Psychol.

[35] He J, Liu HH (2014). Encouraging college students' to seek professional psychological help. J Coll Advisor.

[36] Tao S, Dong Q, Pratt MW, Hunsberger B, Pancer SM (2000). Social support: relations to coping and adjustment during the transition to university in the People’s Republic of China. J Adolesc Res.

[37] Compas BE, Connor-Smith JK, Saltzman H, Thomsen AH, Wadsworth ME (2001). Coping with stress during childhood and adolescence: problems, progress, and potential in theory and research. Psychol Bull.

[38] Mamun MA, Griffiths MD (2020). A rare case of bangladeshi student suicide by gunshot due to unusual multiple causalities. Asian J Psychiatr.

[39] Li DJ, Ko NY, Chen YL, Wang PW, Lu WH (2020). Covid-19-related factors associated with sleep disturbance and suicidal thoughts among the taiwanese public: a facebook survey. Int J Environ Res Public Health.

[40] Tang W, Hu T, Hu B, Jin C, Wang G, Xie C, Chen S, Xu J (2020). Prevalence and correlates of PTSD and depressive symptoms one month after the outbreak of the COVID-19 epidemic in a sample of home-quarantined Chinese university students. J Affect Disord.

[41] The ministry of education of the people’s republic of China. Notice of the Ministry of Education on Responding to the New Crown Pneumonia Epidemic and Doing a Good Job in the Employment and Entrepreneurship of the 2020 National College Graduates. 2020. Available at: http://www.moe.gov.cn/srcsite/A15/s3265/202003/t20200306_428194.html . Accessed 5 Mar 2020.

[42] Li X, Lv S, Liu L, Chen R, Zhao J (2020). Covid-19 in guangdong: immediate perceptions and psychological impact on 304,167 college students. Front Psychol.

[43] Bontcheva K, Gorrell G, Wessels B. Social media and information overload: survey results. Computer Science. 2013.

[44] Li YZ (2016). Stress life events and suicide ideation among high school students: a mediated moderation model. Chin J Clin Psychol.

[45] Caballero Domínguez C, Jiménez M, Campo-Arias A (2020). Suicide risk during the lockdown due to coronavirus disease (COVID-19) in Colombia. Death Stud.

[46] Kim Y, Baik SY, Jin MJ, Choi KH, Lee SH. The mediating effect of embitterment on the relationships between anxiety, depression, and suicidality. Psychother Psychosom. 2020;1–3. 10.1159/000506647 10.1159/000506647 32208392

[47] Zhu XL, Liu D, Yan F, Qu W, Fan HZ, Zhao YL, Wang ZR, Tan YL, Ren YP, Tan SP (2020). Psychological status of school students and employees during the COVID-19 epidemic. Chin Ment Health J.

[48] Duffy ME, Twenge JM, Joiner TE (2019). Trends in mood and anxiety symptoms and suicide-related outcomes among u.s. undergraduates, 2007–2018: evidence from two national surveys. J Adolesc Health.

[49] Wang C, Zhao H (2020). The impact of covid-19 on anxiety in chinese university students. Front Psychol.

[50] Li J, Chen SY, Zhu L, Zhang WD (2011). Investigation on knowledge of prevention and control and psychological anxiety about h1n1 influenza among college students in a university, zhengzhou. Modern Prev Med.

[51] Chen L, Fu CJ, Li WH (2004). Study of the relationship between coping style and anxiety of universities students during the SARS. Chin J Health Psychol.

[52] Rajappa K, Gallagher M, Miranda R (2012). Emotion dysregulation and vulnerability to suicidal ideation and attempts. Cogn Ther Res.

[53] Killgore WDS, Cloonan SA, Taylor EC, Fernandez F, Grandner MA, Dailey NS (2020). Suicidal ideation during the COVID-19 pandemic: the role of insomnia. Psychiatry Res.

[54] Ji K, Liu L, Wang P, Huang LL, Xie D, Lu YJ (2020). Sleep quality and mental health of college students studying at home. Modern Prev Med.

[55] Sooyeon S, Hyun K, Yang HC, Rin CE, Ku LS, Chol S (2013). Longitudinal course of depression scores with and without insomnia in non-depressed individuals: a 6-year follow-up longitudinal study in a korean cohort. Sleep.

[56] Bozzay ML, Karver MS, Verona E (2016). Linking insomnia and suicide ideation in college females: the role of socio-cognitive variables and depressive symptoms in suicide risk. J Affec Disord.

[57] Na PJ, Yaramala SR, Kim JA, Kim H, Goes FS, Zandi PP, Voort JL, Sutor B, Croarkin P, Bobo WV (2018). The PHQ-9 Item 9 based screening for suicide risk: a validation study of the Patient Health Questionnaire (PHQ)−9 Item 9 with the Columbia Suicide Severity Rating Scale (C-SSRS). J Affec Disord.

